# Pan-Bcl-2 Inhibitor AT-101 Enhances Tumor Cell Killing by EGFR Targeted T Cells

**DOI:** 10.1371/journal.pone.0047520

**Published:** 2012-11-19

**Authors:** Archana Thakur, Lawrence G. Lum, Dana Schalk, Asfar Azmi, Sanjeev Banerjee, Fazlul H. Sarkar, Ramzi Mohommad

**Affiliations:** 1 Departments of Oncology and Medicine, Karmanos Cancer Institute, Wayne State University, Detroit, Michigan, United States of America; 2 Department of Pathology, Karmanos Cancer Institute, Wayne State University, Detroit, Michigan, United States of America; 3 Immunology and Microbiology, Karmanos Cancer Institute, Wayne State University, Detroit, Michigan, United States of America; University of Nebraska Medical Center, United States of America

## Abstract

Pancreatic cancer is a deadly disease and has the worst prognosis among almost all cancers and is in dire need of new and improved therapeutic strategies. Conditioning of tumor cells with chemotherapeutic drug has been shown to enhance the anti-tumor effects of cancer vaccines and adoptive cell therapy. In this study, we investigated the immunomodulatory effects of pan-Bcl-2 inhibitor AT-101 on pancreatic cancer (PC) cell cytotoxicity by activated T cells (ATC). The effects of AT-101 on cytotoxicity, early apoptosis, and Granzyme B (GrzB) and IFN-γ signaling pathways were evaluated during EGFR bispecific antibody armed ATC (aATC)-mediated killing of L3.6pl and MiaPaCa-2 PC cells pre-sensitized with AT-101. We found that pretreatment of tumor cells with AT-101 enhanced susceptibility of L3.6pl and MiaPaCa-2 tumor cells to ATC and aATC-mediated cytotoxicity, which was in part mediated via enhanced release of cytolytic granule GrzB from ATC and aATC. AT-101-sensitized L3.6pl cells showed up-regulation of IFN-γ-mediated induction in the phosphorylation of Ser^727^-Stat1 (pS^727^-Stat1), and IFN-γ induced dephosphorylation of phospho-Tyr^705^-Stat3 (pY^705^-Stat3). Priming (conditioning) of PC cells with AT-101 can significantly enhance the anti-tumor activity of EGFRBi armed ATC through increased IFN-γ induced activation of pS^727^-Stat1 and inhibition of pY^705^-Stat3 phosphorylation, and resulting in increased ratio of pro-apoptotic to anti-apoptotic proteins. Our results verify enhanced cytotoxicity after a novel chemotherapy conditioning strategy against PC that warrants further *in vivo* and clinical investigations.

## Introduction

Although chemotherapy is considered myelo- and immunosuppressive [1], the combination of a number of chemotherapeutic regimens have been shown to enhance the anti-tumor effects of cancer vaccines and adoptive cell therapy [2]–[7]. Pretreatment of tumors with chemotherapy has not only shown improved anti-tumor efficacy of immunotherapy, but has also shown success in breaking self-tolerance by eliminating MDSC and attenuating the tumor suppressive environment leading to enhanced anti-tumor immunity [8]–[11]. On the other hand, tumor sensitization with immunotherapy prior to chemotherapy, the “chemocentric chemoimmunotherapy” approach has also shown profound enhancement of the cytotoxic effect of chemotherapeutic drugs [12].

Among the emerging members of small molecule pan-Bcl-2 inhibitors, AT-101(*R*-(-)-gossypol acetic acid), a polyphenolic compound, has been shown to inhibit Bcl-2 by acting as a BH3 mimetic which disrupts the heterodimerization of Bcl-2, Mcl-1, and Bcl-X_L_ with proapoptotic family members [13]–[16]. As a single agent in multiple Phase I and Phase II trials, AT-101 exhibited cytoreductive activity in chronic lymphocytic leukemia (CLL), non-Hodgkin's Lymphoma (NHL), and prostate cancer patients [17]–[19]. While in other Phase I/II studies in solid tumors, AT-101 either as a single agent or in combination therapy failed to show clinical efficacy mainly due to dose related toxicities [20], [21]. We hypothesized that combining pan-Bcl-2 inhibitor AT-101 at a suboptimal concentration with targeted activated T-cells may offer a greater treatment efficacy.

Pancreatic cancer (PC) remains a deadly and by far incurable disease killing over 33,000 US citizens annually, and five year survival is less than 5% [22]. Standard chemotherapy involving gemcitabine has negligible impact on the dismal statistics while neo adjuvant therapies involving combination regimens such as FOLFURINOX have shown only marginal benefits [23]. Thus, novel therapies are urgently needed for the treatment of pancreatic cancer. Small molecule inhibitors that target the intracellular tyrosine kinase signaling pathways of EGFR, such as gefitinib (Iressa®) or erlotinib (Tarceva®) have been tested in clinical trials without major impact on the disease in spite of the fact that EGFR is over-expressed in 30–50% of pancreatic cancer [24]–[26]. However, targeting EGFR through bispecific antibody (EGFRBi) armed activated T-cells (aATC) offers a novel and non-toxic approach that exploits EGFR over-expression independent of EGFR activation state and/or mutations. We compared the anti-tumor effects of combining a suboptimal concentration of AT-101 with EGFRBi armed ATC or the effect of each individually. Our data show that pre-sensitization of tumor cells with a suboptimal concentration of AT-101 can significantly enhance the anti-tumor activity of EGFRBi armed ATC, and thus this strategy could be useful for designing novel therapies for the treatment of PC.

## Materials and Methods

### Cell Lines and Reagents

The human pancreatic cancer (PC) cell lines (MiaPaCa-2, and CoLo-357) were obtained from American Type Culture Collection (Rockville, MD). The human pancreatic L3.6pl cells were established from Colo-357 cells by injecting them into the pancreas of nude mice [27]. These cell lines were maintained in RPMI-1640 or DMEM culture media (Lonza Inc., Allendale, NJ) supplemented with 10% FBS (Lonza Inc.), 2 mM L-glutamine (Invitrogen, Carlsbad, CA), 50 units/ml penicillin, and 50 µg/ml streptomycin (Invitrogen). Pan-Bcl-2 inhibitor, AT-101 was a gift from Shaomeng Wang (Ann Arbor Michigan). Antibodies for flow cytometry were purchased from BD Biosciences and Cell Signaling Technologies.

### Expansion and Generation of ATC and Production of Anti-OKT3×Anti-EGFR Bispecific Antibodies

Human PBMC were isolated from the heparinized whole blood of normal healthy donors using lymphocyte separation solution. The Wayne State University Institutional Review Board approved research protocols for blood collection from normal healthy donors. All normal donors signed consent forms. Activated T cells (ATC) from PBMC were expanded using 20 ng/ml of OKT3 and 100 IU/ml of IL-2 for 14 days at a concentration of 1–2×10^6^ PBMC/ml in RPMI-1640 supplemented with 10% FBS. Bispecific Antibodies (BiAb) were produced by chemical heteroconjugation of OKT3 (a murine IgG2_a_ anti-CD3 monoclonal antibody, Ortho Biotech, Horsham, PA) and Erbitux (a chimeric anti-EGFR IgG_1_, Bristol-Myers Squibb, Princeton, NJ) as described earlier [28]. ATC were armed with anti-CD3×anti-EGFR (EGFRBi) bispecific antibodies (aATC) following a previously optimized concentration of BiAb [29] (50 ng/10^6^ ATC) for 30 minutes prior to its use in experiments.

### Cytotoxicity Assay

Cytotoxicity testing was performed using chromium release assay using ^51^Cr labeled L3.6pl and MiaPaCa-2 cells as described earlier [30]. Briefly, tumor cells were seeded in 96-well plates at 40,000 cells/well in a volume of 100 µl. Cells were allowed to adhere overnight before 0.5 µM and 1 µM AT-101 (these concentrations were chosen based on our dose titration experiments documenting minimal cytotoxicity) was added and incubated for 24–72 h. Following priming, cells were washed to remove AT-101 before labeling with ^51^Cr. EGFRBi armed ATC were added at 10∶1 effector to target (E∶T) ratio for additional overnight incubation with ^51^Cr labeled targets. AT-101 alone, unarmed ATC or armed ATC alone served as controls at each time point. Experiments were repeated three times in quadruplicate wells to ensure the reproducibility.

### Detection of Early Apoptosis

For detection of apoptosis, untreated and 1 µM AT-101 treated PC cells were incubated for 24 h followed by 1–4 h of incubation with EGFRBi armed ATC at 10∶1 E∶T. Cells were then stained with Annexin V-FITC (BD Biosciences) and the viability dye 7-AAD. The proportion of Annexin V-positive cells was measured within the population of tumor cells by flow cytometry. All cells prepared for flow cytometry were analyzed using FACSCalibur instrumentation (BD) and FlowJo software.

### Flow Cytometric Quantification of Markers for Apoptotic Pathway

Untreated and 1 µM AT-101 treated L3.6pl and MiaPaCa-2 cells were incubated for 24 h, followed by 1–4 h of incubation with EGFRBi armed ATC at 10∶1 E∶T. AT-101 alone, unarmed ATC or armed ATC alone served as controls at each time point. For intracellular staining, brefeldin A, a protein transport inhibitor, was added during incubation of effectors with targets. At the end of incubation, ATC or aATC were removed by washing twice, adherent tumor cells were removed by trypsinization. The cells were washed, fixed, and permeabilized with Fix/Perm solution (BD Biosciences) and then stained for 60 minutes on ice with fluorochrome-labeled anti-phospho-Stat1 (Phospho-Stat1, Ser727), anti-phospho-Stat3 (Phospho-Stat3, Tyr705), anti-Bcl-X_L_, anti-Bax, anti-phospho-Bad (Phospho-Bad,Ser112) rabbit monoclonal antibodies (Cell Signaling Technology) followed by flow cytometric analysis. For surface staining, the cells were blocked with 5% human serum in PBS for 30 minutes and labeled with mouse monoclonal anti-CD3-FITC or anti-EpCAM-APC antibodies (BD Biosciences) for 30 minutes followed by washing and fixing in 2% paraformaldehyde in PBS and analyzed by flow cytometry.

### Staining for IFN-γ, Granzyme B and Degranulation Markers

PC cells, L3.6pl and MiaPaCa-2, were treated with 1 µM of AT-101 for 24 h prior to the assay. The cells from both untreated and AT-101-treated groups were incubated for 1–4 h with EGFRBi armed ATC at 10∶1 E∶T. Target cells were stained for surface and intracellular granzyme (GrzB) and degranulation markers, CD107a and CD107b. In a separate experiment degranulation of ATC and aATC following stimulation was stopped by GolgiStop. ATC or aATC were then stained for intracellular GrzB and CD107a/b using Perm/Fix kit and corresponding antibodies from BD Biosciences. The percentage of GrzB and CD107a/b positive cells was calculated for tumor cells, ATC, and aATC in each group.

### IFN-γ ELISA of Culture Supernatants

Cytokines were quantitated in culture supernatants collected from untreated or AT-101 treated cultures in the presence or absence of ATC or aATC using ELISA kit as per manufacturer's instruction (R&D Systems).

### Statistical Analysis

Quantitative data are presented as the mean of at least three or more independent experiments ± standard deviation. A one-way ANOVA was used to determine whether there were statistically significant differences within each experiment. Differences between groups were tested via an unpaired, two-tailed *t* test.

## Results

### Enhanced Anti-Tumor Effect of Armed ATC (aATC) in AT-101 Primed Tumor Cells

**Figure 1:**
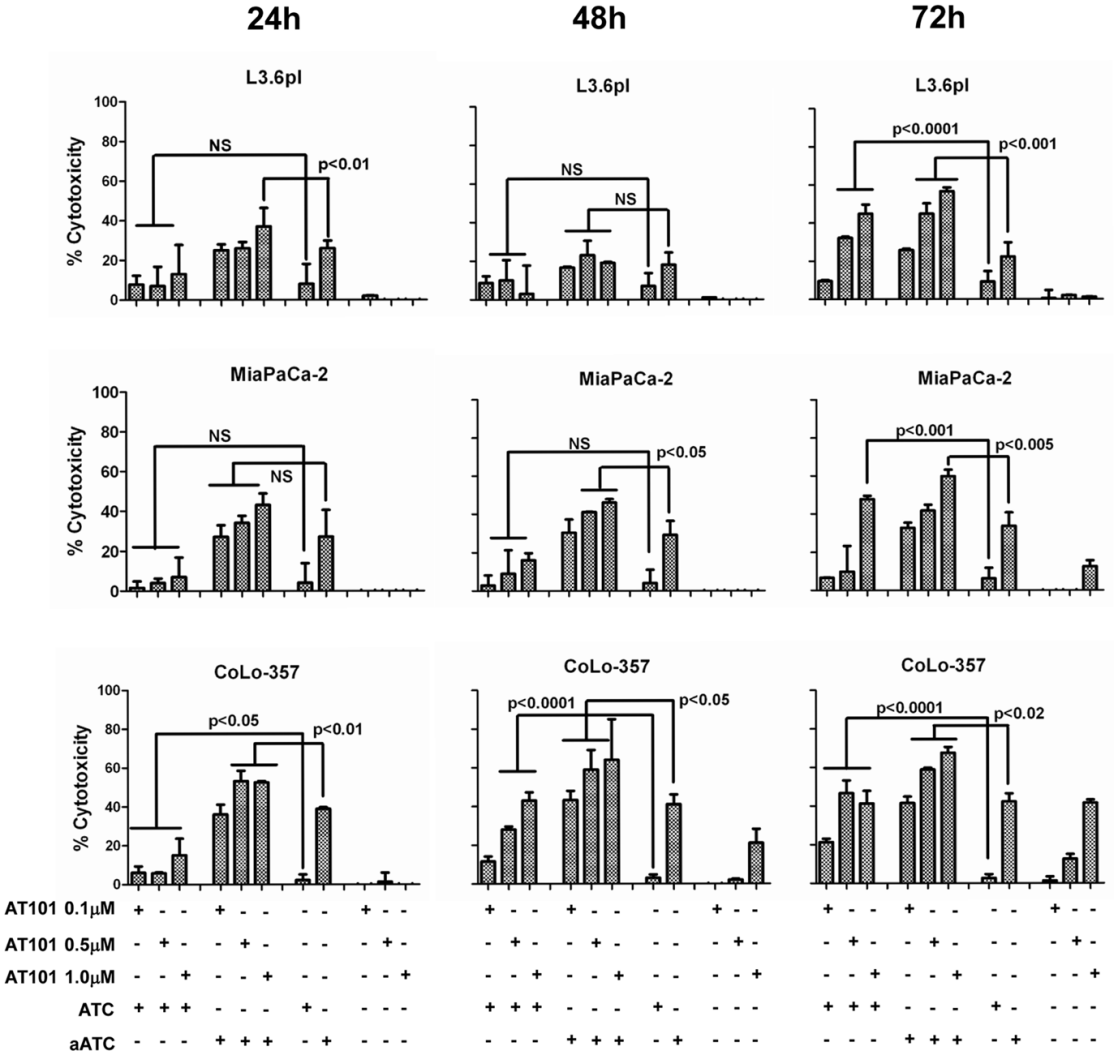
Kinetics of cytotoxicity induced by ATC, aATC, pan Bcl-2 inhibitor-AT-101, and the combination of AT-101 with immunotherapy approach. Chromium release assay was performed in triplicate in three pancreatic cancer (PC) cell lines (L3.6pl, MiaPaCa-2 and CoLo FG) at 24, 48 and 72 h of treatment with AT-101 followed by 18 h incubation with ATC or aATC at 10∶1 E/T ratio. Incubation of target cells with AT-101 alone at indicated time points, ATC and aATC alone for 18 h served as controls.

**Figure 2:**
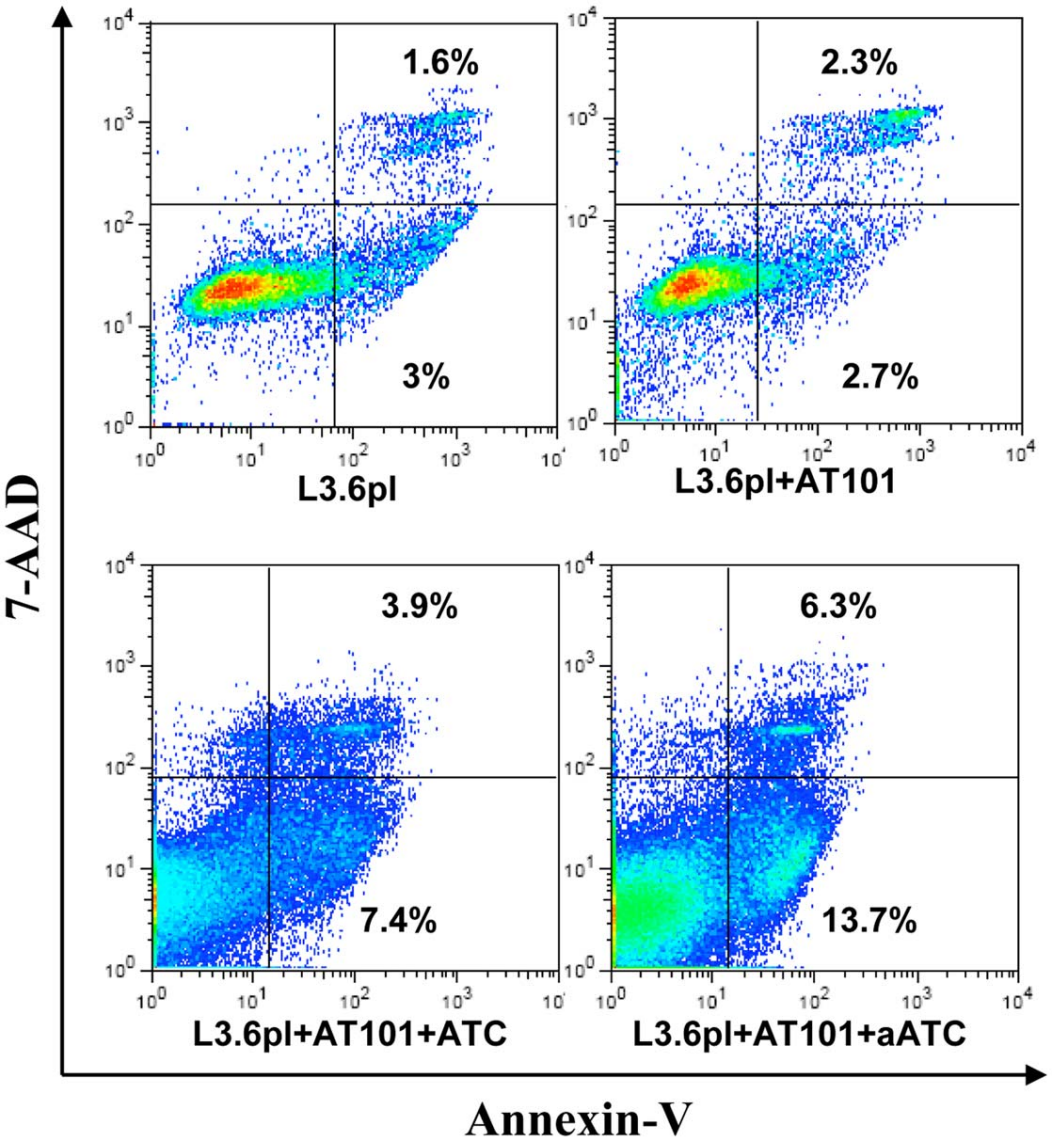
Early apoptosis was detected in MiaPaCa-2 and L3.6pl cells after a 24 h pretreatment with AT-101 and a subsequent 4 h incubation with ATC or aATC at 10∶1 E/T. The treated cells were stained with AnnexinV-FITC and 7-AAD and the proportion of Annexin V positive cells was gated on tumor cells by flow cytometry.

We investigated whether the chemotherapy agent can affect the susceptibility of tumor cells to the lytic effect of aATC. We treated three PC cell lines with 0.1, 0.5, 1.0 and 5.0 µM concentrations of AT-101 for 24, 48 and 72 h followed by overnight incubation with ATC or EGFRBi armed ATC (aATC) and measured the cytotoxicity by ^51^Cr release assay. Both ATC and EGFRBi armed ATC showed significantly enhanced cytotoxicity in AT-101 primed PC cell lines in a priming time and dose dependent manner. Cytotoxicity by ATC or EFGRBi armed ATC increased significantly (40–70%) after priming of L3.6pl, MiaPaCa-2 and CoLo-357 cells for 72 h with AT-101 at 0.5 µM and 1 µM concentrations, suggesting an additive cytotoxic effect of combination therapy (**Figure 1**). There was no difference in the cytotoxicity observed in 0.1 µM AT-101 treated or untreated cells, while the 5.0 µM concentration was technically limiting due to the fragility of the primed tumor cells to withstand ^51^Cr labeling and multiple washing steps. Since AT-101 did not induce cytotoxicity in the first 24 h at 1 µM concentration, we chose 1 µM AT-101 treatment for 24 h followed by ATC or aATC incubation for 1–4 h in all our subsequent experiments. Considerable apoptosis was observed in L3.6pl cells treated with ATC or aATC after 4 h treatment (**Figure 2**).

### AT-101 Enhanced Cytotoxic Activity of ATC and aATC

**Figure 3:**
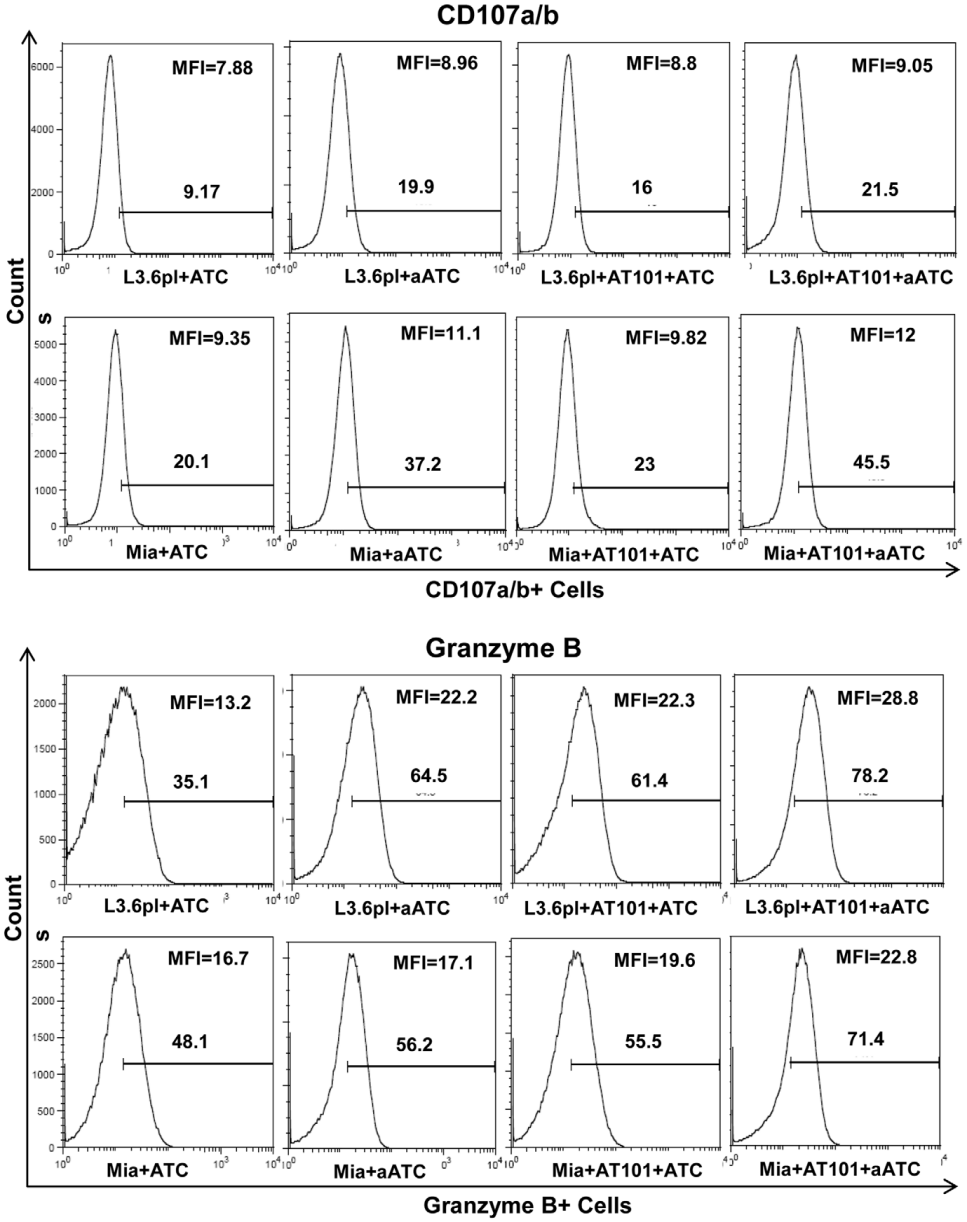
Mechanism of apoptosis induced by aATC in AT-101 primed tumor cells. MiaPaCa-2 (or Mia) and L3.6pl cells were either pretreated with AT-101 for 24 h or left untreated followed by additional incubation with ATC or aATC for 4 h in the presence of GolgiStop and staining for intracellular CD107a and b. Y-axis shows the counts and the X-axis of each histogram shows the percentage of fluorochrome positive cells.

Earlier we have shown that the perforin/granzyme pathway plays a prominent role in ATC and aATC-mediated cytotoxic effects [30]. We therefore examined the effect of 1 µM AT-101 in a 4 h assay on degranulation (CD107a and b) and GrzB release in ATC or aATC when stimulated with target cells. No difference in the proportion of CD107a^+^ and CD107b^+^ cells was observed during incubation of ATC or aATC with 1 µM AT-101 treated or untreated L3.6pl and MiaPaCa-2 for 4 h at 10∶1 E/T ratio. However, the number of GrzB^+^ cells increased in both ATC and aATC (p<0.05) when incubated with AT-101 treated tumor cells for 4 h (**Figures 3** **and** **4**). Mean fluorescence intensity (MFI) for CD107a/b and GrzB also increased in both ATC and aATC (p<0.04) in the presence of AT-101 treated L3.6pl cells (**Figures 3** **and** **4**), suggesting that chemotherapy sensitizes tumor cells for enhanced aATC-mediated cytotoxicity via up-regulation of these molecules.

**Figure 4:**
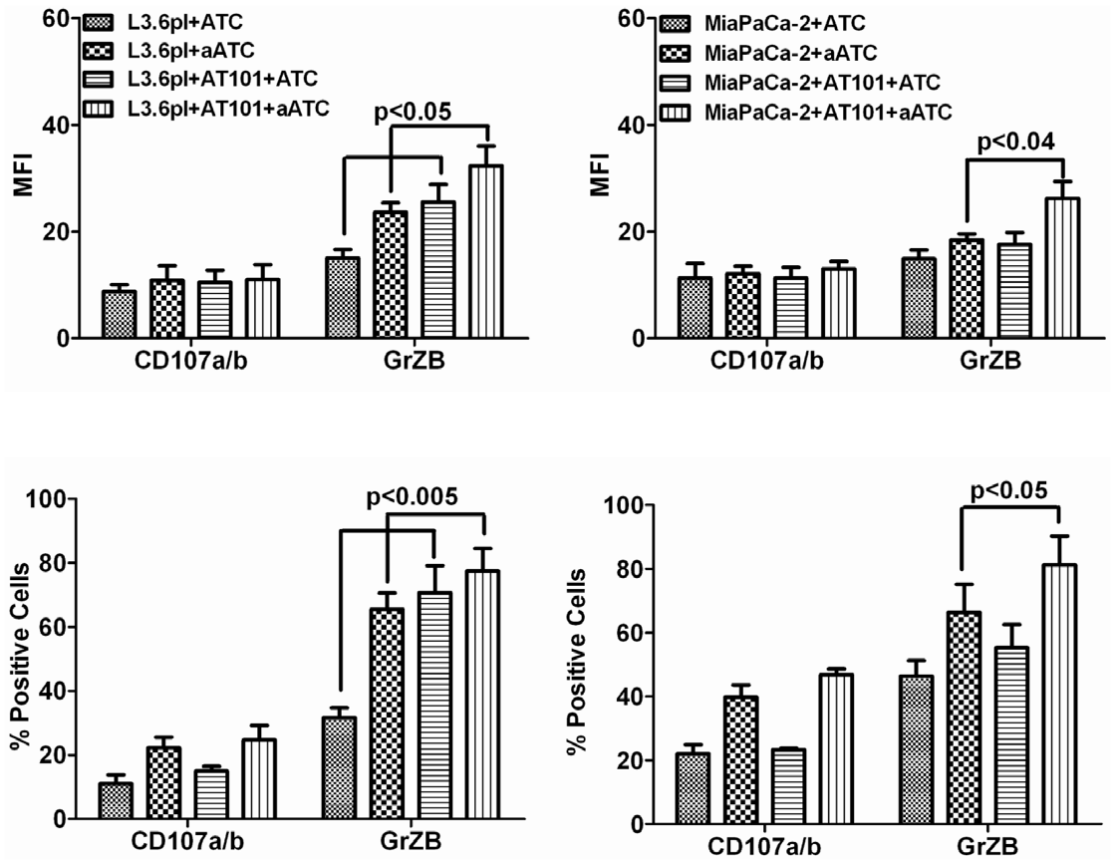
Intracellular staining for Granzyme B by flow cytometry. MiaPaCa-2 (or Mia) and L3.6pl cells were either pretreated with AT-101 for 24 h or left untreated followed by additional incubation with ATC or aATC for 4 h in the presence of GolgiStop and staining for intracellular Granzyme B. Y-axis shows the counts and the X-axis of each histogram shows the percentage of fluorochrome positive cells.

### Enhanced IFN-γ Expression in aATC During Interaction with AT-101 Treated Tumor Cells

**Figure 5:**
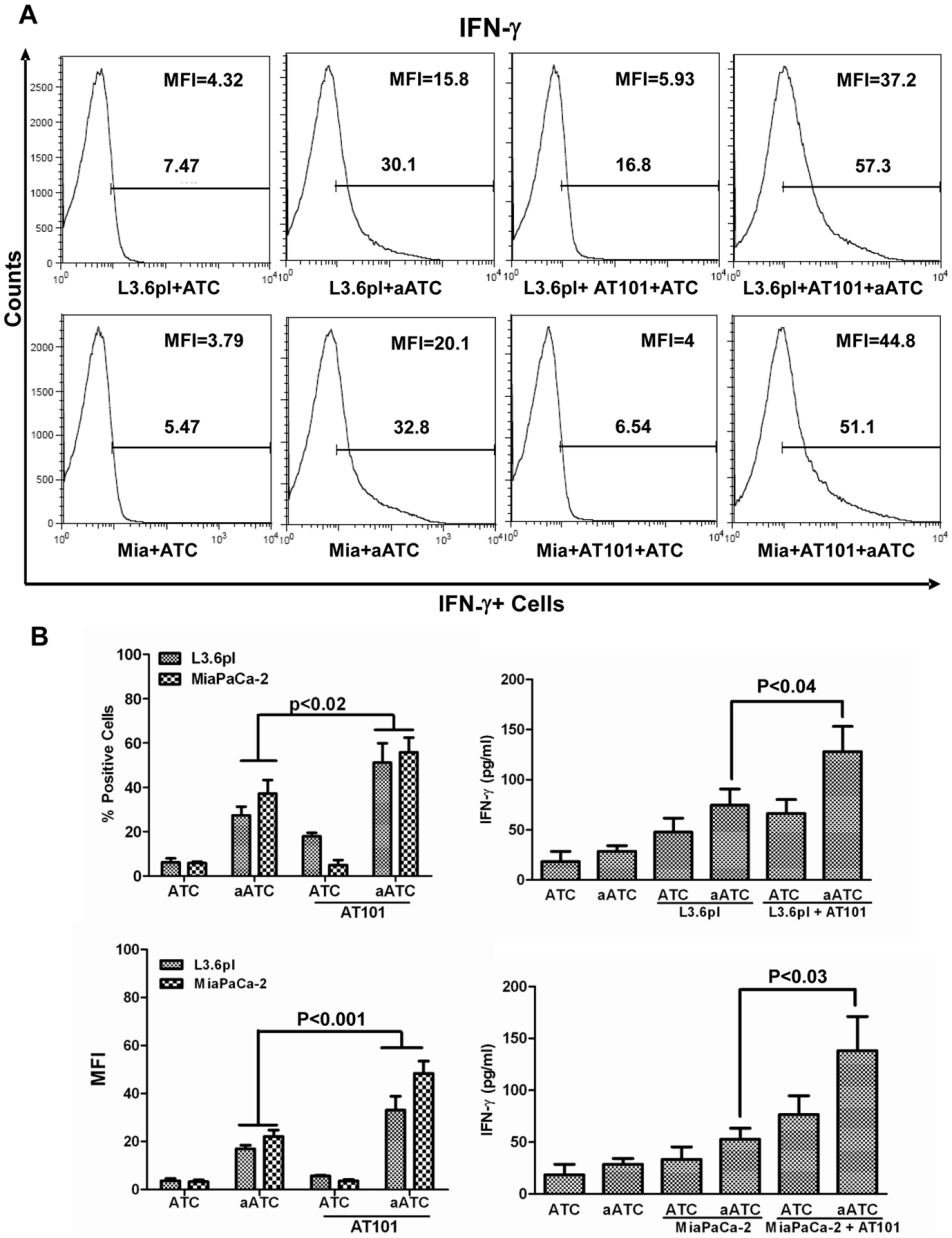
Intracellular staining for IFN-γ by flow cytometry. MiaPaCa-2 (or Mia) and L3.6pl cells were either pretreated with AT-101 for 24 h or left untreated and a subsequent 4 h incubation with ATC or aATC in the presence of GolgiStop followed by staining for intracellular IFN-γ by flow cytometry. Y-axis shows the counts and the X-axis of each histogram shows the percentage of fluorochrome positive cells. **B**). Culture supernatants from AT-101 sensitized PC cells co-cultured with ATC or aATC in the absence of GolgiStop were used to detect the secreted IFN-γ in the culture supernatants, and data are presented as mean ± SD from one representative experiment. Y-axis shows the counts and the X-axis of each histogram shows the percentage of fluorochrome positive cells.

Our previous studies have shown that high levels of IFN-γ are produced during aATC-mediated killing of target cells [31], [32]. We examined whether IFN-γ participates in AT-101 sensitized tumor cell killing by ATC and aATC, by staining for intracellular IFN-γ in ATC and aATC, and by measuring IFN-γ in culture supernatant after 4 h co-culture with L3.6pl and MiaPaCa-2 cells either pre-sensitized with AT-101 or left untreated. Intracellular staining for IFN-γ showed marked increase in aATC co-cultured with L3.6pl or MiaPaCa-2 cells compared to those treated with ATC (3–7 fold increase in MFI) regardless of AT-101 sensitization. Incubation of aATC with AT-101 treated cells showed significantly higher numbers of positive cells (1.5–2-fold; p<0.02) as well as 2 fold increase in MFI (p<0.001) over aATC incubated with untreated L3.6pl or MiaPaCa-2 cells (**Figures 5A and 5B**). Similarly, IFN-γ levels increased significantly (p<0.04) in the culture supernatant of both cell lines sensitized with AT-101 and co-cultured with aATC for 4 h. Upregulation of IFN-γ provides further support that ATC/aATC interaction with AT-101 sensitized target cells enhance effector functions of ATC and aATC.

### AT-101 Priming Makes Tumor Cells Susceptible for Cytolytic Activity by aATC

**Figure 6:**
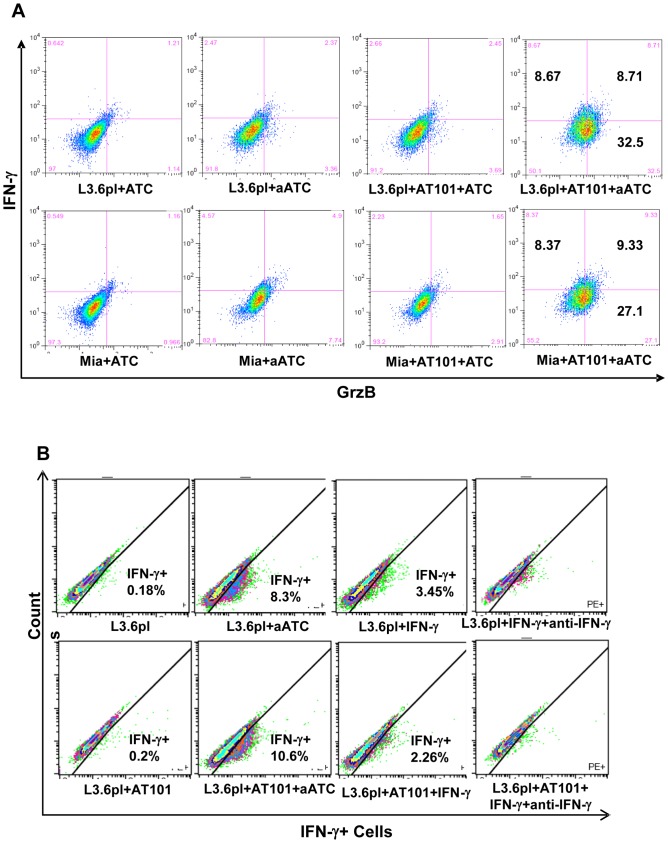
Evidence of enhanced GrzB-mediated killing of in AT-101 sensitized tumor cells. MiaPaCa-2 (Mia) and L3.6pl cells were either pretreated with AT-101 for 24 h or left untreated and a subsequent 4 h incubation with ATC or aATC followed by staining for surface IFN-γ and intracellular Grz B. Proportion of IFN-γ and GrzB positive cells was gated on tumor cells by flow cytometry. **B**) Shows surface staining for IFN-γ by flow cytometry in L3.6pl cells incubated with 10 ng/ml recombinant human IFN-γ protein with or without 10 µg/ml anti-IFN-γ antibodies in the presence or absence of AT-101. Y-axis shows the counts and the X-axis of each histogram shows the percentage of fluorochrome positive cells.

Next, we tested whether AT-101 sensitized L3.6pl and MiaPaCa-2 cells become more susceptible for aATC-mediated killing. We stained AT-101 treated and untreated tumor cells after 4 h incubation with ATC or aATC for surface IFN-γ and intracellular GrzB. The proportion of IFN-γ positive tumor cells increased ∼2–3 fold in both L3.6pl and MiaPaCa-2 cells when tumor cells were pretreated with AT-101 compared with untreated tumor cells (**Figure 6A**). The proportion of GrzB positive tumor cells increased more than 2–7 fold in L3.6pl and MiaPaCa-2 cells after priming with AT-101 compared with untreated tumor cells incubated with ATC or aATC (**Figure 6A**). Since 4 h co-culture of aATC with tumor cells showed surface staining of IFN-γ on tumor cells, we sought to determine the specificity of IFN-γ binding by adding 10 ng/ml recombinant human IFN-γ protein alone or with anti-IFN-γ antibody (10 µg/ml) in L3.6pl or MiaPaCa-2 cells with or without AT-101 for 1 h followed by washing and surface staining with anti-IFN-γ-PE. Data show that 1 h incubation with either IFN-γ or aATC resulted in IFN-γ binding to the cell surface; however, the IFN-γ staining was higher in the presence of aATC in AT-101 primed cells (**Figure 6B**). These data suggest that tumor cell priming with 1 µM AT-101 may enhance IFN-γ and GrzB-mediated cytotoxicity of tumor cells induced by ATC or aATC.

### aATC Immunotherapy Induced Stat1 Phosphorylation and Inhibited Stat3 Phosphorylation in AT-101 Sensitized PC Cells

**Figure 7:**
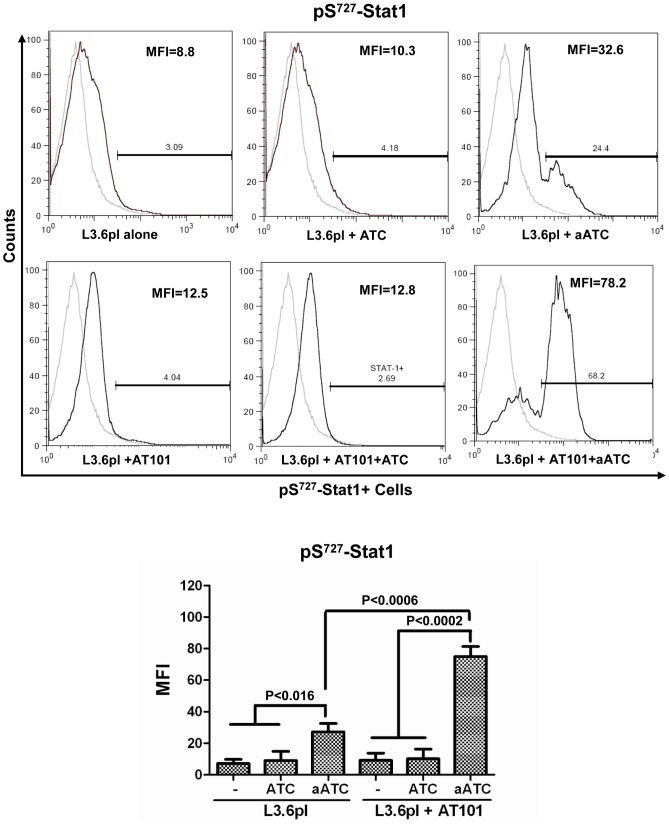
The combination of pan Bcl-2 inhibitor AT-101 with aATC immunotherapy induces Stat1 phosphorylation. L3.6pl cells were either pretreated with AT-101 for 24 h or left untreated followed by additional incubation with ATC or aATC for 4 h and staining for intracellular phospho-Stat1. Proportion of Stat1 positive cells and MFI were gated on tumor cells. Top panel show histogram overlays representing isotype control and phospho-Stat1 positive tumor cells. Bottom panel shows the graphic representation of MFI under the indicated experimental conditions.

**Figure 8:**
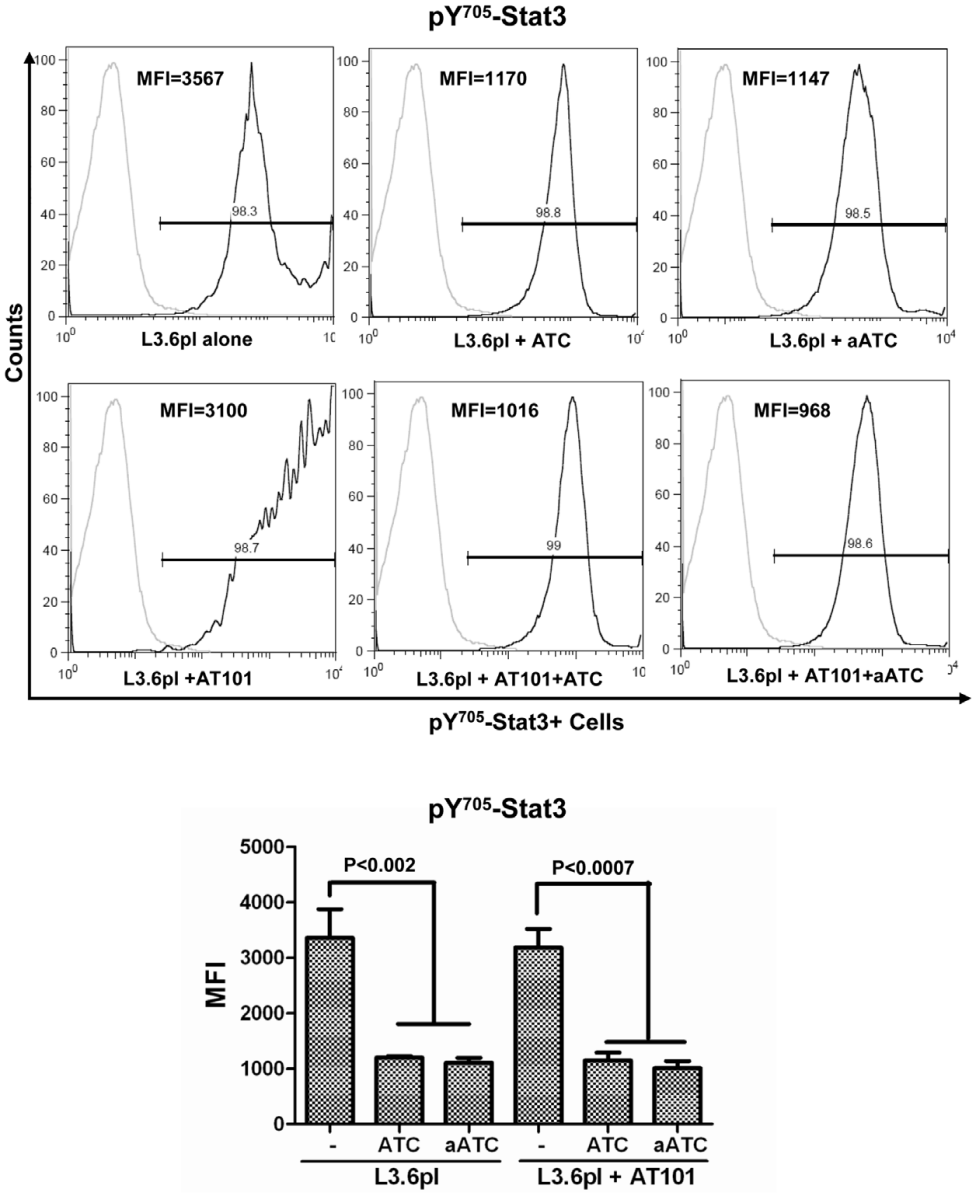
The combination of pan Bcl-2 inhibitor AT-101 with aATC immunotherapy inhibits Stat3 phosphorylation. L3.6pl cells that were either pretreated with AT-101 for 24 h or left untreated followed by additional incubation with ATC or aATC for 4 h. Proportion of Stat3 positive cells and MFI were gated on tumor cells. Top panel show histogram overlays representing isotype control and phosphor-Stat3 positive tumor cells. Bottom panel shows the graphic representation of MFI under the indicated experimental conditions.

The signal transducers and activators of transcription (Stat) factors function as downstream effectors of cytokine and growth factor receptor signaling [33], [34]. Constitutive Stat3 activation can lead to resistance to apoptosis [35]. We show that high levels of IFN-γ are produced during aATC-mediated targeted killing of tumor cells, thus we reasoned that IFN-γ may induce apoptosis by inhibiting Stat3 phosphorylation and inducing Stat1 phosphorylation. Our data show that pS^727^-Stat1 positive cells (p<0.002) and their MFI were significantly higher in the presence aATC in AT-101 treated (p<0.0002) or untreated (p<0.016) L3.6pl cells compared to tumor cells alone, tumor cells treated with AT-101 with or without ATC. Both pS^727^-Stat1 positive cells and MFI were significantly higher (p<0.0006) when aATC were incubated with AT-101 treated L3.6pl cells (**Figure 7**). As expected, MFI of pY^705^-Stat3 positive cells were significantly reduced in the presence of ATC (p<0.002) or aATC (p<0.0007) regardless of AT-101 priming of L3.6pl cells. Phospho-Stat3 levels were also reduced when L3.6pl cells were treated with AT-101; however, the difference was not statistically significant (**Figure 8**). Total Stat1 and Stat3 levels were not statistically significant among various treatment conditions (data not shown).

**Figure 9:**
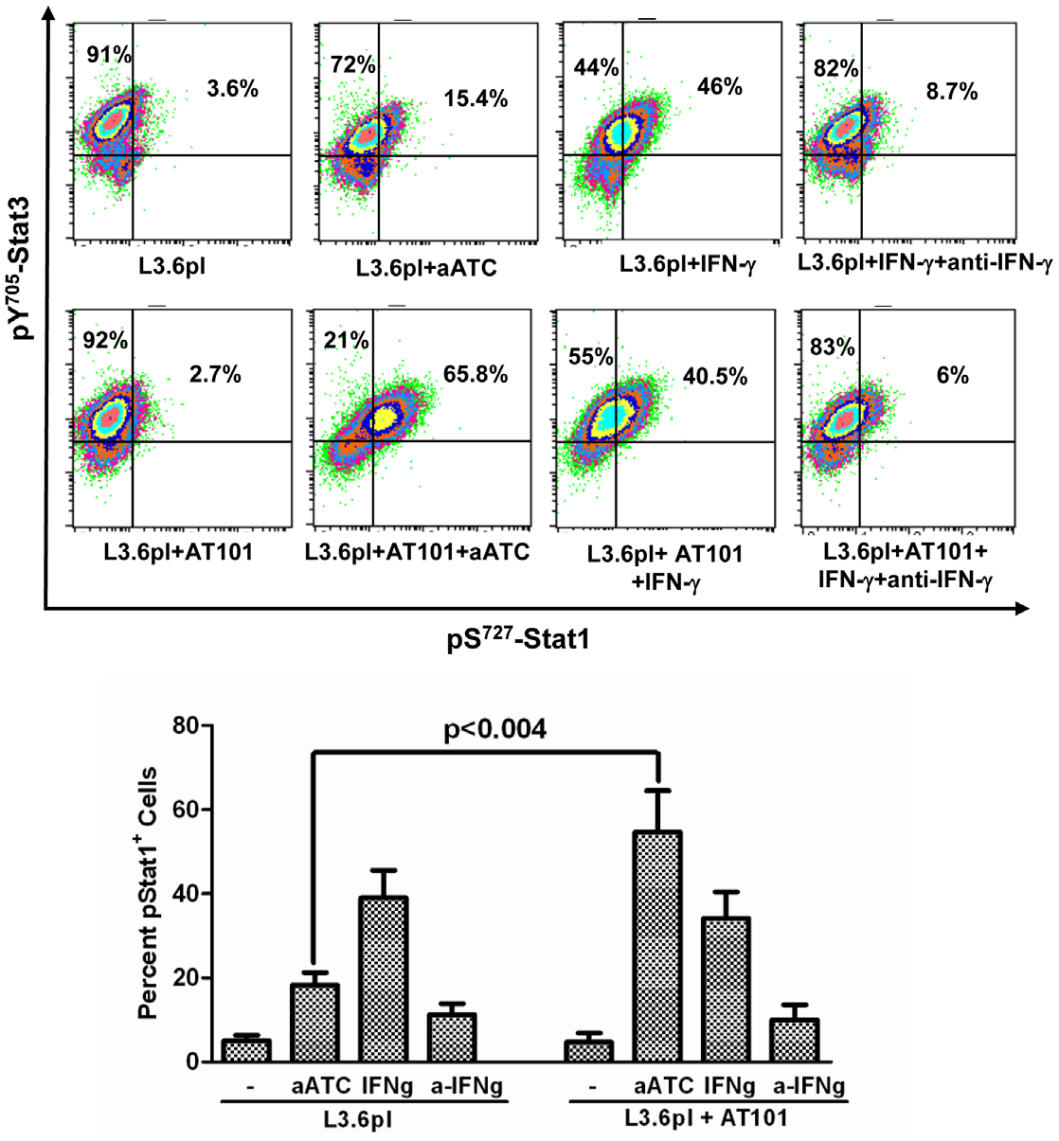
Shows induction of pS^727^-Stat1 in L3.6pl cells incubated with 10 ng/ml recombinant human IFN-γ protein with or without 10 µg/ml anti-IFN-γ antibodies in the presence or absence of AT-101 (*p<0.05; **p<0.005; ***p<0.0005).

To confirm whether the induction of pS^727^-Stat1 is mediated by IFN-γ signaling, **a**) we added 10 ng/ml recombinant human IFN- γ protein in L3.6pl and MiaPaCa-2 cells with or without AT-101 followed by intracellular staining for pS^727^-Stat1 and pY^705^-Stat3 to assess the direct effect of IFN-γ on pS^727^-Stat1 expression, **b**) in another setting, experiments were done in the presence of 10 ng/ml recombinant human IFN-γ protein and anti-IFN-γ antibody (10 µg/ml) with or without AT-101 followed by intracellular staining for pS^727^-Stat1 and pY^705^-Stat3. Interestingly, IFN-γ containing L3.6pl and MiaPaCa-2 cells showed a remarkable increase in the proportion of pS^727^-Stat1^+^ cells that was decreased in the presence of anti-IFN-γ antibodies and this was not affected by AT-101 (**Figures 9** **and** **10**).

**Figure 10:**
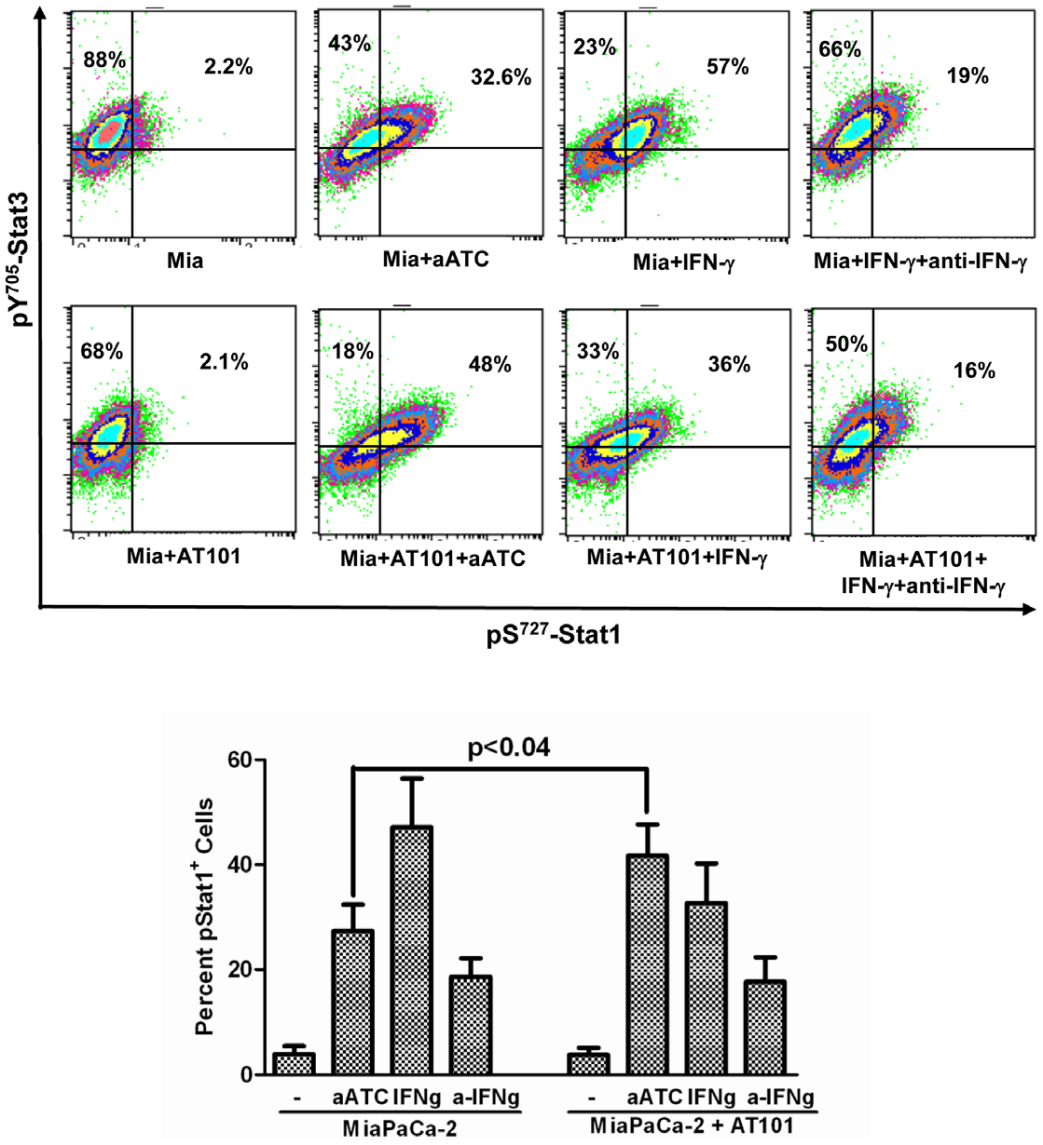
Shows induction of pS^727^-Stat1 in MiaPaCa-2 cells incubated with 10 ng/ml recombinant human IFN-γ protein with or without 10 µg/ml anti-IFN-γ antibodies in the presence or absence of AT-101 (*p<0.05; **p<0.005; ***p<0.0005).

### aATC Immunotherapy Suppresses Anti-Apoptotic Bcl-X_L_ and Induces Pro-Apoptotic Proteins Bax and Bad in AT-101 Sensitized Tumor Cells

**Figure 11:**
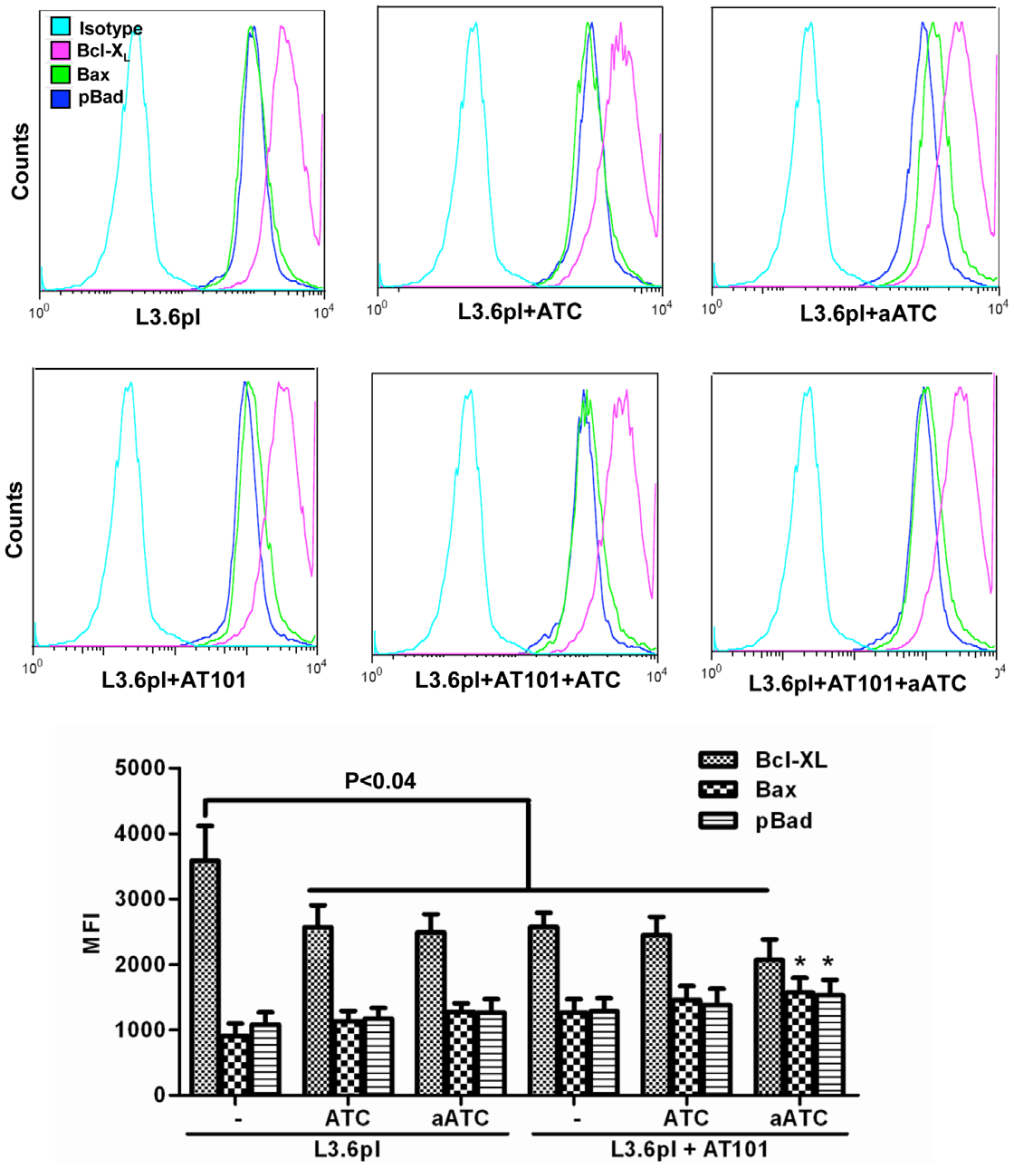
The combination of pan Bcl-2 inhibitor AT-101 with aATC immunotherapy induces pro-apoptotic proteins and inhibits the expression of anti-apoptotic proteins. L3.6pl cells were either pretreated with AT-101 for 24 h or left untreated followed by additional incubation with ATC or aATC for 4 h and before intracellular staining for pBad, Bax and Bcl-X_L_. Top panel show histogram overlays representing isotype control, phosphor-Bad, Bax and Bcl-X_L_ positive cells. Proportion of phosphor-Bad, Bax and Bcl-X_L_ positive cells and MFI were gated on tumor cells. Bottom panel shows the graphic representation of MFI under the indicated experimental conditions.

**Figure 12:**
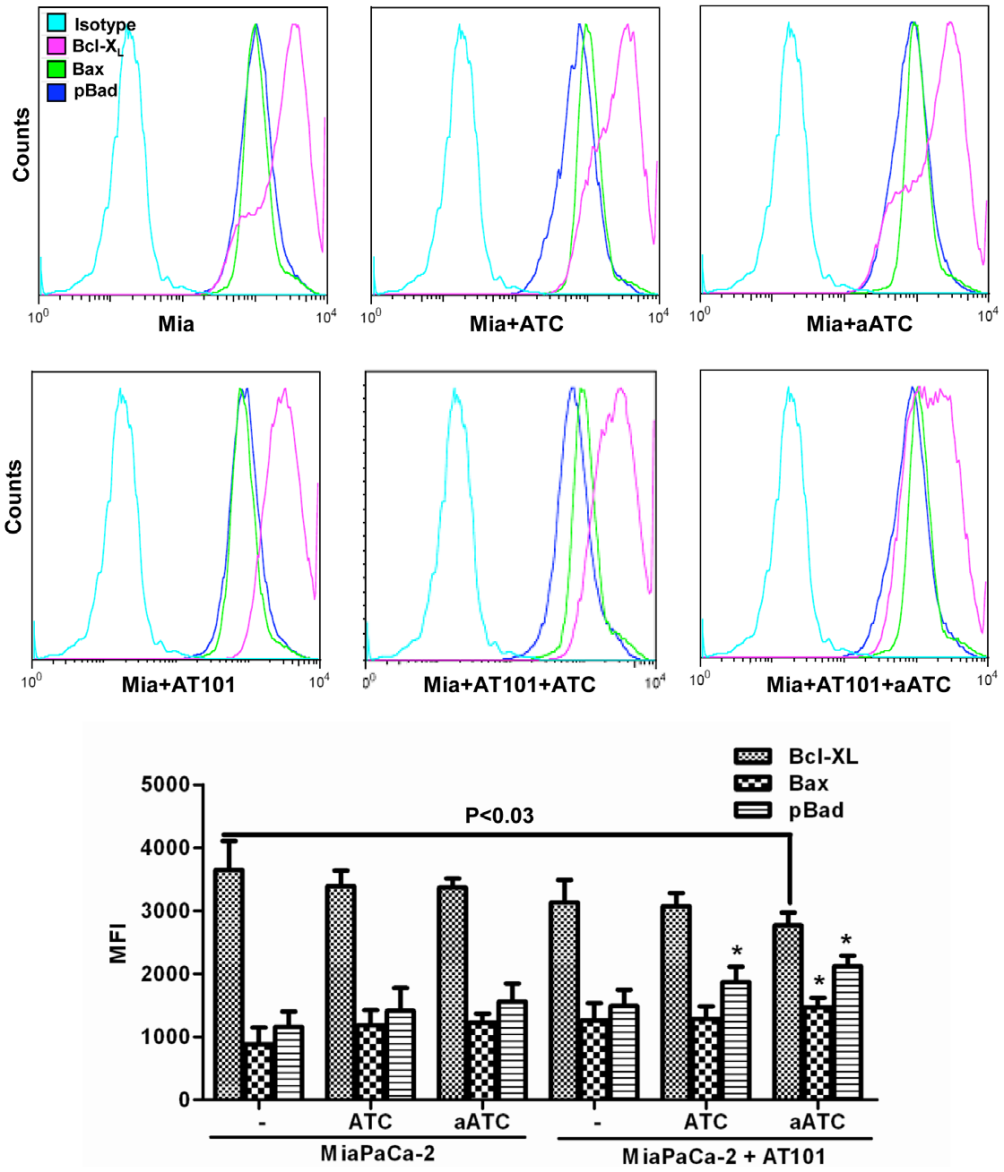
Shows intracellular staining for pBad, Bax and Bcl-X_L_ in MiaPaCa-2 cells were either pretreated with AT-101 for 24 h or left untreated followed by incubation with ATC or aATC before staining. Top panel show histogram overlays representing isotype control, phosphor-Bad, Bax and Bcl-X_L_ positive cells. Proportion of phosphor-Bad, Bax and Bcl-X_L_ positive cells and MFI were gated on tumor cells. Bottom panel shows the graphic representation of MFI under the indicated experimental conditions.

**Figure 13:**
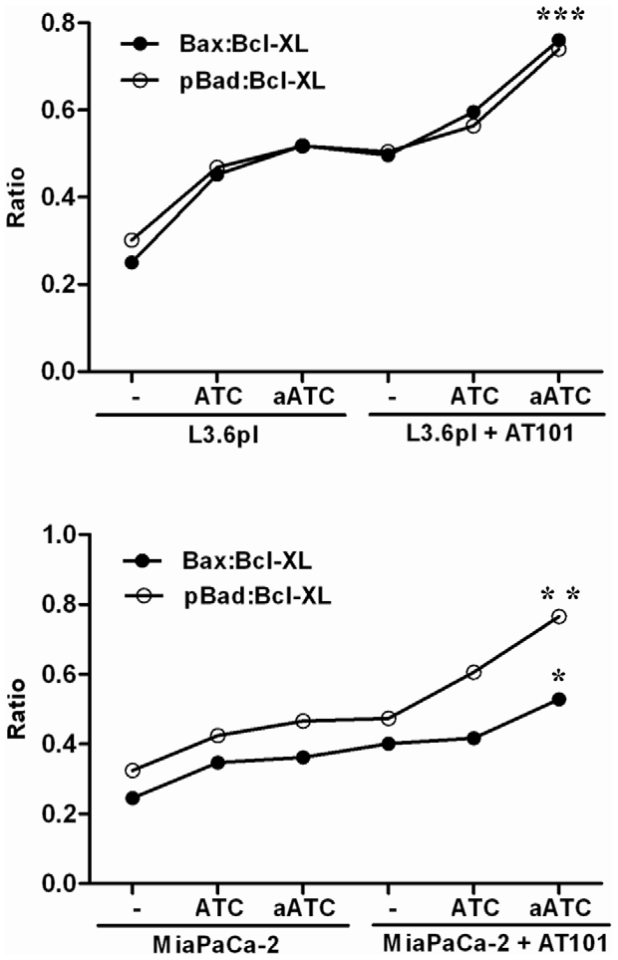
Shows the ratio of p-Bad: Bcl-X_L_ and Bax: Bcl-X_L_ under the indicated experimental conditions. Ratio of p-Bad: Bcl-X_L_ and Bax: Bcl-X_L_ showed a significant difference between AT-101 treated and untreated L3.6pl and MiaPaCa-2 cells in the presence of aATC. Representative data from two experiments with the same results are shown.

Since ATC and aATC treated tumor cells showed significant dephosphorylation of pY^705^-Stat3 and increased phosphorylation of pS^727^-Stat1, we next examined the effect of Stat3 dephosphorylation on its target anti-apoptotic gene Bcl-X_L_, and Stat1 activation on its target pro-apoptotic genes Bax and Bad. We stained Bcl-X_L_, pBad and Bax proteins intracellularly and analyzed for percent positive cells and relative mean fluorescence intensities. Our data demonstrate a significant reduction in the MFI of Bcl-X_L_, (L3.6pl, p<0.04; MiaPaca-2, P<0.03) and an increase in pro-apoptotic pS^112^-Bad (L3.6pl, p<0.05; MiaPaca-2, P<0.03) and Bax (L3.6pl, p<0.04; MiaPaca-2, P<0.05) expression in both AT-101 sensitized L3.6pl cells (**Figure 11**) and MiaPaCa-2 cells (**Figure 12**) treated with EGFRBi armed ATC. Upper panels show histogram overlays representing isotype control, Bcl-X_L_, Bax and p-Bad positive cells gated on tumor cells (**Figure 11** **and** **12**). Interestingly, ratios of both pBad ∶Bcl-X_L_ and Bax∶Bcl-X_L_ MFI were also noticeably higher (more death) in both cell lines, L3.6pl (pBad ∶Bcl-X_L_, p<0.0001; Bax∶Bcl-X_L_, p<0.0001) and MiaPaCa-2 (pBad ∶Bcl-X_L_, p<0.005; Bax∶Bcl-X_L_, p<0.05) treated with EGFRBi armed ATC after 24 h AT-101 sensitization (**Figure 13**).

## Discussion

**Figure 14:**
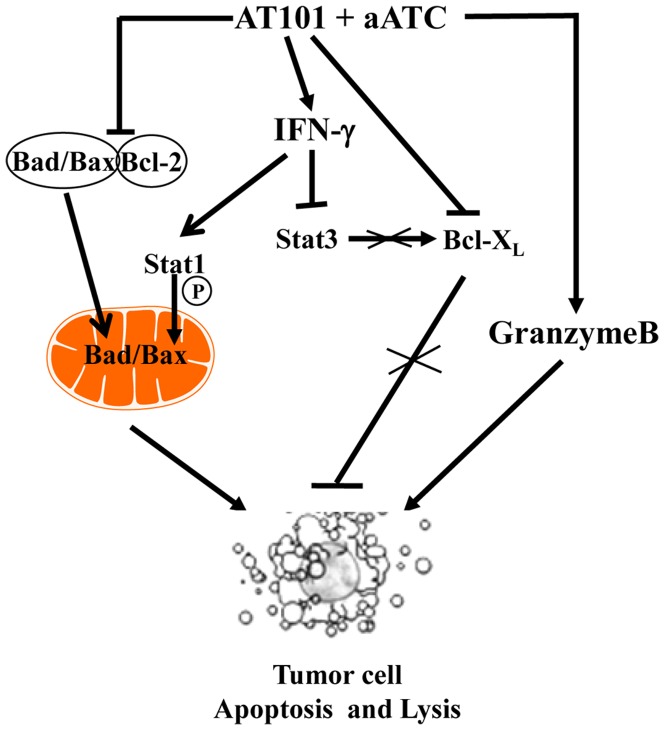
Schematic diagram showing the mechanism of EGFRBi armed ATC-mediated killing of AT-101 sensitized tumor cells. AT-101 a Pan-Bcl-2 inhibitor binds to the BH3 binding domain of Bcl-2 and disrupts its interaction with pro-apoptotic family members such as Bax and Bad. Free Bax and Bad re-localizes into the mitochondria where they initiate the mitochondrial apoptosis. Our results demonstrate that tumor cell stimulated aATC can produce IFN-γ that in turn can phosphorylate Stat1. pStat activation results in Bad/Bax mitochondrial localization and induction of apoptosis. IFN-γ itself is a suppressor of anti-apoptotic Bcl-X_L_.

PC is among the deadliest of all malignancies and urgently requires new therapeutic approaches. Pretreatment of tumor cells with small molecule inhibitors or chemotherapeutic drugs has been shown to sensitize tumor cells for enhanced CTL responses by altering the expression levels of key apoptosis regulators [36]–[38]. Using this principle, we have for the first time investigated the effect of combination therapy where PC cells were pretreated with suboptimal concentrations of AT-101 followed by incubation with ATC and aATC-mediated cytotoxicity of tumor targets. We observed that 1) sensitization of tumor cells with AT-101 enhanced ATC and aATC-mediated cytotoxicity in L3.6pl, MiaPaCa-2 and CoLo-357 cells; 2) enhanced GrzB uptake of tumor cells; 3) enhanced IFN-γ induced Stat1 phosphorylation and Stat3 dephosphorylation, which in turn inhibited Bcl-X_L_ and induced expression of pBad and Bax (**Figure 14**). EGFRBi armed ATC-mediated apoptosis in AT-101 sensitized L3.6pl and MiaPaCa-2 cells may be facilitated by increased expression of pBad and Bax and partially through repressing Bcl-X_L_ expression.

ATC are known to induce cytotoxicity via Fas/FasL or perforin/granzyme pathways; however, in previous studies we have shown that perforin/granzyme is the predominant pathway of aATC-mediated killing [39]. During effector-target interaction, the pore forming protein perforin is released by activated T cell (ATC) and facilitates the delivery of serine proteases GrzA and GrzB to the target cell cytoplasm and nucleus, where they deliver the apoptotic hits through independent pathways [40]–[42]. First, we asked whether interaction of AT-101 sensitized PC cells with ATC or aATC can enhance perforin/granzymes-mediated cytotoxicity of tumor cells by ATC or aATC. Induction of early apoptosis was determined by Annexin V/7-AAD, which showed no difference between untreated tumor cells and tumor cells treated with 1 µM AT-101 (24 h). These data suggest that a suboptimal concentration of AT-101 could not induce apoptosis within 24 h exposure but was sufficient to cause rapid induction of apoptosis following 4 h incubation with ATC or aATC.

We examined the changes in degranulation markers CD107a/b and cytolytic granule GrzB following a 4 h incubation of ATC or aATC with AT-101 treated tumor cells at 10∶1 E/T. ATC and aATC incubated with AT-101 treated tumor cells showed increased numbers of GrzB positive cells as well as increased expression compared to ATC or aATC incubated with untreated tumor cells. CD107a/b did not show much change in percent positivity or expression in ATC and aATC incubated with AT-101 treated cells compared to untreated tumor cells. Interestingly, an increased percentage of GrzB positive tumor cells were seen after tumor cells were sensitized with AT-101 compared to untreated tumor cells in the presence of ATC and aATC, suggesting that AT-101 sensitization enhances penetration of cytolytic granules into the tumor cells. These results are consistent with other studies showing increased penetration of GrzB during cytotoxicity with antigen specific CTL activity in chemotherapy sensitized tumor cells [36].

Earlier, we have shown that stimulation of ATC or aATC with target cells induces production of IFN-γ. IFN-γ-induced phosphorylation of Stat1 and dephosphorylation of Stat3 play essential roles in anti-proliferative, pro-apoptotic effects of IFN-γ [33], [43], [44]. Since constitutive phosphorylation of Stat3 has been detected in a variety of human cancers including pancreatic tumors and PC cell lines [33], [45], [46], we examined whether ATC or aATC can induce increased apoptosis of AT-101 sensitized tumor cells via IFN-γ/Stat pathways. Our results show a significantly reduced expression (MFI) of pY^705^-Stat3 in the presence of ATC or aATC regardless of AT-101 sensitization, suggesting that ATC and aATC alone are potent inhibitors of Stat3 phosphorylation in the tumor cells. Interestingly, pS^727^-Stat1 expression and percent positive cells both increased significantly in AT-101 sensitized tumor cells in the presence of aATC. In addition, a significant correlation was found between IFN-γ and Stat1 expression.

Since Stat1 and Stat3 show opposing patterns that promote or inhibit apoptosis, respectively, we examined the expression of anti-apoptotic Bcl-X_L_ and pro-apoptotic pBad and Bax proteins in AT-101 sensitized tumor cells in the presence of ATC or aATC. Studies have shown that IFN-γ-induced dephosphorylation of Stat3 could induce apoptosis [33] through down-regulation of Bcl-2, Bcl-X_L_, and Mcl-1, and up-regulation of Bax [47]. AT-101 treated L3.6pl and MiaPaCa-2 cells showed reduced MFI for Bcl-X_L_ and increased MFI for pS^112^-Bad and Bax compared to untreated L3.6pl and MiaPaCa-2 cells in the presence of ATC or aATC; however, the difference was not statistically significant. Intriguingly, the ratio of pBad∶Bcl-X_L_ and Bax∶Bcl-X_L_ under the same conditions showed a significant difference between AT-101 treated and untreated L3.6pl and MiaPaCa-2 cells. Constitutively high levels of Bcl-2 or Bcl-X_L_ have been associated with drug resistance and more aggressive phenotype in both liquid and solid tumors [48], [49]. Expression of Bcl-X_L_ in the NCI 60 cell line panel strongly correlated with resistance to most chemotherapy agents [50]. Cellular fate between apoptosis and survival depends upon the balance between proapoptotic and antiapoptotic protein levels. High levels of Bcl-X_L_ and low levels of Bax provide a survival advantage and vice versa. Del Poeta et al. (2003) showed that the ratio of Bax/Bcl-2 MFI predicted the outcome in acute myeloid leukemia (AML) [51]. Lower Bax/Bcl-2 levels were correlated with poor-risk cytogenetics; and a longer overall survival (OS) and disease-free survival (DFS) observed in patients with higher Bax/Bcl-2 levels. Another study in B-CLL showed that enhanced Bcl-2/Bax ratio contributes to B-CLL survival [52]. These results are consistent with our current findings.

While combination of targeted therapies with chemotherapeutic drugs has been tested against PC, this is the first study, to the best of our knowledge, to report that priming of tumor cells with highly specific Bcl-2 inhibitor enhances cytotoxic activity armed ATC. In summary, we have shown that the combination of Bcl-2 inhibitor AT-101 (conditioning of PC cells) with targeted immunotherapy using EGFRBi armed ATC offers an attractive therapeutic approach that can lead to reduced toxicity but potent anti-tumor activity. Priming of tumor cells with suboptimal concentration for short duration can significantly enhance anti-tumor activity of EGFRBi armed ATC through the inhibition of Stat3 and activation of Stat1. These proof-of-concept studies provide rationale to design future strategies for the treatment of pancreatic cancer and warrant further clinical investigations against deadly PC.
